# Implications of bond disorder in a *S*=1 kagome lattice

**DOI:** 10.1038/s41598-018-23054-6

**Published:** 2018-03-16

**Authors:** Jamie L. Manson, Jamie Brambleby, Paul A. Goddard, Peter M. Spurgeon, Jacqueline A. Villa, Junjie Liu, Saman Ghannadzadeh, Francesca Foronda, John Singleton, Tom Lancaster, Stewart J. Clark, Iorwerth O. Thomas, Fan Xiao, Robert C. Williams, Francis L. Pratt, Stephen J. Blundell, Craig V. Topping, Christopher Baines, Charles Campana, Bruce Noll

**Affiliations:** 1 Department of Chemistry and Biochemistry, Eastern Washington University, Cheney, WA 99004 United States; 2 Department of Physics, University of Warwick, Coventry, CV4 7AL United Kingdom; 3 Clarendon Laboratory, Department of Physics, University of Oxford, Oxford, OX1 3PU United Kingdom; 4 National High Magnetic Field Laboratory, Los Alamos National Laboratory, Los Alamos, NM 87545 United States; 5 Center for Materials Physics, Durham University, Durham, DH1 3LE United Kingdom; 6 STFC, ISIS Pulsed Muon Facility, Rutherford-Appleton Laboratory, Chilton, Oxfordshire, OX11 0QX United Kingdom; 7 Paul Scherrer Institut, Laboratory for Muon-Spin Spectroscopy, CH-5232 Villigen PSI, Switzerland; 8 Bruker AXS, Inc, Madison, WI 53711 United States

## Abstract

Strong hydrogen bonds such as F···H···F offer new strategies to fabricate molecular architectures exhibiting novel structures and properties. Along these lines and, to potentially realize hydrogen-bond mediated superexchange interactions in a frustrated material, we synthesized [H_2_F]_2_[Ni_3_F_6_(Fpy)_12_][SbF_6_]_2_ (Fpy = 3-fluoropyridine). It was found that positionally-disordered H_2_F^+^ ions link neutral NiF_2_(Fpy)_4_ moieties into a kagome lattice with perfect 3-fold rotational symmetry. Detailed magnetic investigations combined with density-functional theory (DFT) revealed weak antiferromagnetic interactions (*J* ~ 0.4 K) and a large positive-*D* of 8.3 K with *m*_s_ = 0 lying below *m*_s_ = ±1. The observed weak magnetic coupling is attributed to bond-disorder of the H_2_F^+^ ions which leads to disrupted Ni-F···H-F-H···F-Ni exchange pathways. Despite this result, we argue that networks such as this may be a way forward in designing tunable materials with varying degrees of frustration.

## Introduction

Long-range magnetic order (LRO) in low-dimensional materials is often the result of a delicate balance of competing interactions. This balance can be perturbed in a number of ways. Typically, the effect of introducing random disorder into a system of interacting spins is to suppress the onset of an ordered state. However, the inherent structural configuration of some magnetic lattices (e.g. triangular, hexagonal, kagome) gives rise to a geometric frustration of the dominant exchange interactions, which itself precludes LRO, leading to a large ground-state degeneracy and the possibility of spin-liquid behavior^1–4^. In general, introducing structural disorder into such a system will act to lift the frustration and restore LRO. The complex interplay between these three phenomena (order, disorder and frustration) is an area of considerable current interest^5–9^. In the present work we set out to synthesize an ideally frustrated kagome lattice of *S* = 1 spins in a tunable molecule-based crystal. Unexpectedly, the material that self-assembles exhibits an unusual randomized network in which one-in-three magnetic exchange pathways is slightly distorted. This random structural disorder lifts the effect of magnetic frustration and should promote the onset of LRO. However, the surprising additional consequence is that the small distortion acts to suppress the size of the effective exchange energies, impeding LRO down to milli-Kelvin temperatures.

Among the possible design elements to self-assemble molecular structures are strong charge-assisted hydrogen bonds (O-H···O, O-H···F and F-H···F)^10,11^. These interactions have proven fruitful to realize materials sensitive to external stimuli such as high pressure^12–14^ or high electric fields^15^. Over the past decade, we have focused on the design, synthesis and physical characterization of open-shell transition metal coordination complexes, molecules and polymers, based on Cu(II) (*S* = 1/2), Ni(II) (*S* = 1) and Co(II) (*S* = 3/2) ions that contain the poly-HF adducts HF_2_^−^, H_2_F_3_^−^ and H_3_F_4_^−^ which have been shown, on occasion, to be effective mediators of magnetic exchange interactions^16–22^. These efforts have led to structures spanning all dimensionalities including mononuclear [Cu(dpd)_2_](H_2_F_3_)_2_ (dpd = di-2-pyridyl-methanediol)^16^, quasi-1D [Ni(HF_2_)(3-Clpy)_4_]BF_4_ (Clpy = chloropyridine)^17,18^, quasi-2D Cu(HF_2_)_2_(pyz) (pyz = pyrazine)^19^, and 3D [*M*(HF_2_)(pyz)_2_]*E*F_6_ (*M* = Co, Ni, Cu; *E* = P, Sb)^20–24^ and [CuAg(H_3_F_4_)(pyz)_5_](SbF_6_)_2_^23^. These materials exhibit a variety of complex magnetic phases in which quantum fluctuations act to reduce the size of the ordered magnetic moment or, in extreme cases, suppress long-range order entirely leading to a quantum disordered state.

In the present work, we employ H···F hydrogen bonds to synthesize [H_2_F]_2_[Ni_3_F_6_(Fpy)_12_][SbF_6_]_2_ (Fpy = 3-fluoropyridine) that contains the rare H_2_F^+^ ion. Single crystal X-ray analysis reveals self-assembled NiF_2_(Fpy)_4_ octahedra with Ni(II) centers arranged on the vertices of a perfect 2D kagome lattice. Despite chemical appearances, the H_3_F_4_^−^ bridges that seemingly link these octahedra are actually positionally-disordered H_2_F^+^ cations needed to charge-balance the interlayer SbF_6_^−^ anions. Density-functional theoretical (DFT) calculations indicate ferromagnetic (FM) exchange interactions (*J*) of −8 K spanning fully ordered Ni-F···H-F-H···F-Ni pathways. However, in the real material, our experiments show that the nearest-neighbor interactions between the spins in the kagome layers are very weak over the temperatures probed. No effects of frustration were observed in our experiments and the bulk thermodynamic properties could be largely explained by the single-ion properties of the Ni(II) ion. From theoretical spin-density maps, we can rationalize this behavior in terms of a novel H···F network of randomized broken linkages that introduce a small distortion along one of every three intralayer exchange pathways.

Finally, based on the results described herein, we suggest how the structure-directing ability of H···F bonds presents a viable route towards a true geometrically-frustrated molecular network.

## Results and Discussion

### Crystal structure

The 100 K structure of [H_2_F]_2_[Ni_3_F_6_(Fpy)_12_][SbF_6_]_2_ was determined by single crystal X-ray diffraction (Table 1). The compound crystallizes in the trigonal space group *R* $\bar{3}$. Evidence for the existence of H_2_F^+^ cations comes from the infrared spectrum (see Fig. S1) that shows a broad peak of medium intensity at 1730 cm^−1^. Aside from weak symmetric and asymmetric C-H stretches from Fpy above 3000 cm^−1^, no additional features were observed between 1800 and 3600 cm^−1^. The lack of O-H stretching and bending modes precludes H_2_O or H_3_O^+^ from the structure.

**Table 1:** X-ray crystallographic refinement details for [H_2_F]_2_[Ni_3_F_6_(Fpy)_12_][SbF_6_]_2_.

Chemical formula	C60 H52 F32 N12 Ni3 Sb2
Formula weight (g mol^−1^)	1968.68
Crystal system, space group	Trigonal, *R*-3
Temperature (K)	100(2)
*a*, *b*, *c* (Å), *γ* (°)	14.3617(5), 14.3617(5), 30.9047(11), 120
*V* (Å^3^)	5520.4(4)
*Z*	3
*μ* (mm^−1^)	1.606
Crystal size (mm^3^)	0.355 × 0.237 × 0.146
No. of measured, independent, observed [*F* > 4*σ*(*F*)] refls.	30065, 4281, 3865
*R*_int_	0.0733
*R*[*F* > 4*σ*(*F*)], *R*_all_, *wR*, *S*	0.0327, 0.0358, 0.0921, 1.045
No. of parameters	233
Δ*ρ*_max_, Δ*ρ*_min_ (e Å^−3^)	0.893, −0.752

**Figure 1:**
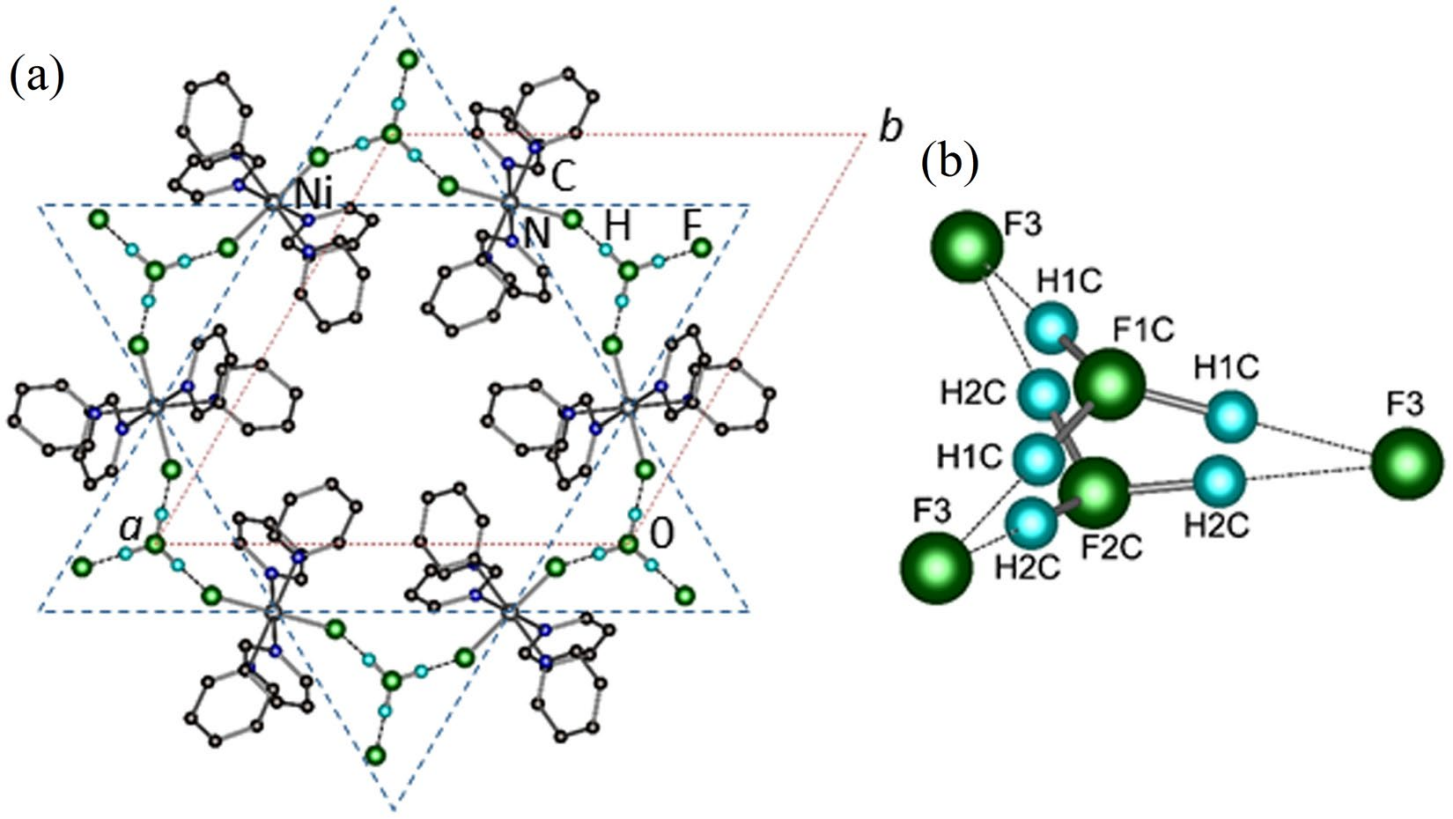
(**a**) Portion of the polymeric structure observed in [H_2_F]_2_[Ni_3_F_6_(Fpy)_12_][SbF_6_]_2_. Ni(II) ions occupy the vertices of a Kagome lattice (blue dashes). The unit cell is highlighted in red. H- and F-atoms from Fpy and SbF_6_^−^ ions are omitted for clarity. (**b**) Sketch of the disordered H_2_F^+^ moiety and adjoining F(3) atoms. Dashed lines delineate longer H···F distances of 1.40 Å. Partial occupancies of H- and F-atoms are given in the text.

The Ni(II) ions occupy axially compressed octahedra comprised of two axial-F ions and four equatorial N atoms donated by Fpy ligands. The coordination sphere is characterized by three bond lengths: Ni-F = 2.000(1) Å, Ni-N = 2 × 2.101(1) and 2 × 2.145(1) Å. The neutral NiF_2_(Fpy)_4_ octahedra occupy the vertices of a 2D kagome lattice (Fig. 1a) with Ni···Ni distances of 7.18 Å. Adjacent kagome layers are well isolated due to intervening SbF_6_^−^ anions. The central-F of the H_2_F^+^ has 3-fold symmetry but is positionally disordered over two sites F1C and F2C (labelling scheme in Fig. 1b) while H1C and H2C are 33% occupied over six positions. Two unique H···F distances of 0.95 and 1.40 Å occur whereas the shortest F···F distance is 2.352(1) Å. The latter distance is typical of poly-HF adducts which are shorter than F···O (≥2.60 Å)^23,25^ and within the sum of van der Waals radii for two fluorine atoms (2.94 Å).

Broadly described, the close proximity of F3 to H_2_F^+^ structurally resembles a H_3_F_4_^−^ anion but with partial occupancies (Fig. 1b). The disordered nature of this species affords random H···F bonds and variable connectivity of NiF_2_(Fpy)_4_ octahedra throughout the 2D kagome network.

Existence of the fluoronium ions H_2_F^+^ and H_3_F_2_^+^ has been established by an X-ray study of separate crystalline phases of [H_2_F][Sb_2_F_11_] and [H_3_F_2_][Sb_2_F_11_]^26^. A terse structural description reported an F···F distance of 2.30(1) Å for the H_3_F_2_^+^ salt whereas H_2_F^+^ exhibited orientational disorder^26^. In either case, none of the protons were located from the X-ray diffraction study nor were any spectroscopic data reported. The presence of H_2_F^+^ in [H_2_F]_2_[Ni_3_F_6_(Fpy)_12_][SbF_6_]_2_ likely arises from the HSbF_6_ precursor^27,28^. Of note is that the occurrence of H_2_F^+^ in a room-temperature stable, solid-state environment is unprecedented.

The unique crystal structure of [H_2_F]_2_[Ni_3_F_6_(Fpy)_12_][SbF_6_]_2_ warranted detailed study by muon-spin relaxation (*μ*^+^SR), electron-spin resonance (ESR), pulsed-field magnetization, heat capacity, magnetic susceptibility and density-functional theory (DFT).

### Magnetic properties

In an applied field (***H***), the Hamiltonian in eq. 1 was used to model the single-ion properties of *S* = 1 Ni(II) in [H_2_F]_2_[Ni_3_F_6_(Fpy)_12_][SbF_6_]_2_,

1 $$ {\mathcal H} =D{\hat{S}}_{z}^{2}+E({\hat{S}}_{x}^{2}-{\hat{S}}_{y}^{2})+{\mu }_{0}{\mu }_{{\rm{B}}}{{\boldsymbol{H}}}^{{\bf{T}}}g\hat{{\boldsymbol{S}}},$$

where ***g*** = diag (*g*_xy_, *g*_xy_, *g*_z_) is the *g*-tensor and ***Ŝ*** = (Ŝ_x_, Ŝ_y_, Ŝ_z_) are the *S* = 1 spin operators^29^. A zero-field splitting (ZFS) lifts the degeneracy of the *m*_s_ = 0 and *m*_s_ = ±1 energy levels^30^, which is resolved into axial (*D*) and rhombic (*E*) components. In general, the local Ni(II) site symmetry dictates whether easy-plane (*D* > 0) or Ising-like (*D* < 0) anisotropy occurs. For the present compound, *D* and *E* were determined to be + 8.3 and 1.21 K, respectively. This *D* is smaller (and of opposite sign) than *D* = −13.6 K (*D*_str_ = −12.3 pm) calculated using the methodology described in ref.^31^.

### Muon-spin relaxation

The results of *μ*^+^SR measurements suggest that no significant change in static or dynamic magnetic properties occur between 0.019 to 10 K (see Fig. S2). This implies that the spin-exchange interactions between Ni(II) ions will be small which is coincident with the absence of long-range order. Thus, the magnetic behavior for *T* ≥ 0.019 K is largely determined by the single-ion properties of the Ni(II) ion.

### Electron-spin resonance

**Figure 2:**
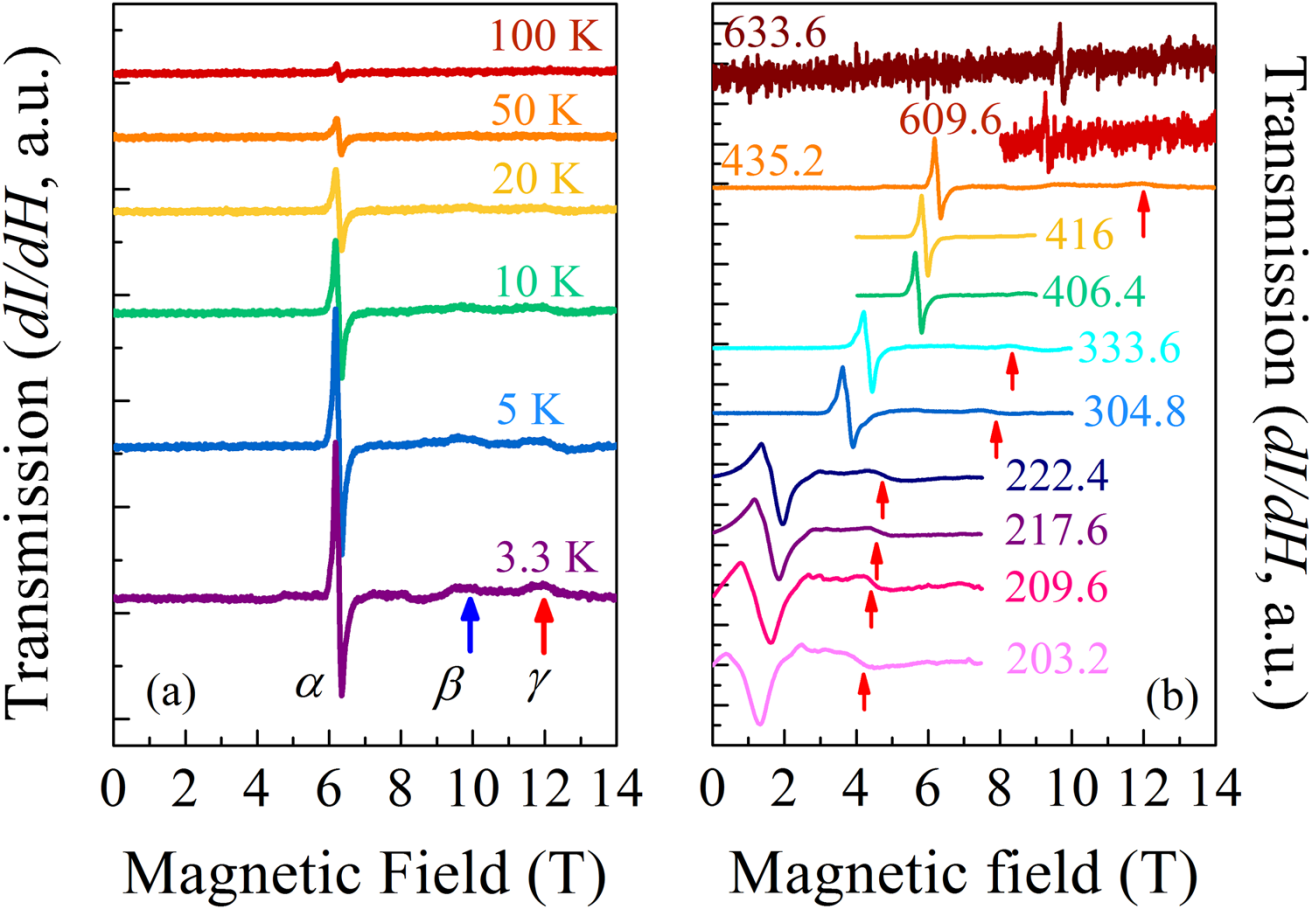
(**a**) Temperature dependence of the powder ESR spectra for [H_2_F]_2_[Ni_3_F_6_(Fpy)_12_][SbF_6_]_2_ collected at 435.2 GHz. Spectra are recorded in the first derivative mode. Besides the main resonance, *α*, at around 6.3 T, two additional small features, *β* and *γ*, emerge at low temperatures as indicated by the blue and red arrows. (**b**) Variable-frequency powder ESR spectra for [H_2_F]_2_[Ni_3_F_6_(Fpy)_12_][SbF_6_]_2_ recorded at 3.5 K for 200 ≤ ν ≤ 635 GHz. Measured frequencies (in GHz) are shown for each spectrum. The *γ* transition is indicated by the red arrows.

Fixed-frequency (*ν*) powder transmission ESR spectra measured at 435.2 GHz for 3.3 ≤ *T* ≤ 100 K (Fig. 2a) show a strong ESR transition (indicated by the derivative shape in differential intensity, d*I*/d*H*) at a resonance field of 6.3 T (labelled *α*). The weak *T*-dependence of the resonance field indicates the transition arises from Ni(II) single-ion spin excitations^32^. Upon cooling, two additional weak features emerge in the powder ESR spectra near 10 and 12 T (*β* and *γ* respectively). The intensity of each transition increases as *T* is reduced, indicating all three features originate from transitions from the ground state within the *S* = 1 multiplet^32^. Absence of spin-correlated behavior is in good agreement with the results of *μ*^+^SR. In *ν*-dependent measurements, the resonance field of the *α* transition is defined by its center where d*I*/d*H* = 0. The field position of the *γ* transition is indicated by the red arrows in Fig. 2b, while the *β* transition is relatively weak and absent from most spectra.

**Figure 3:**
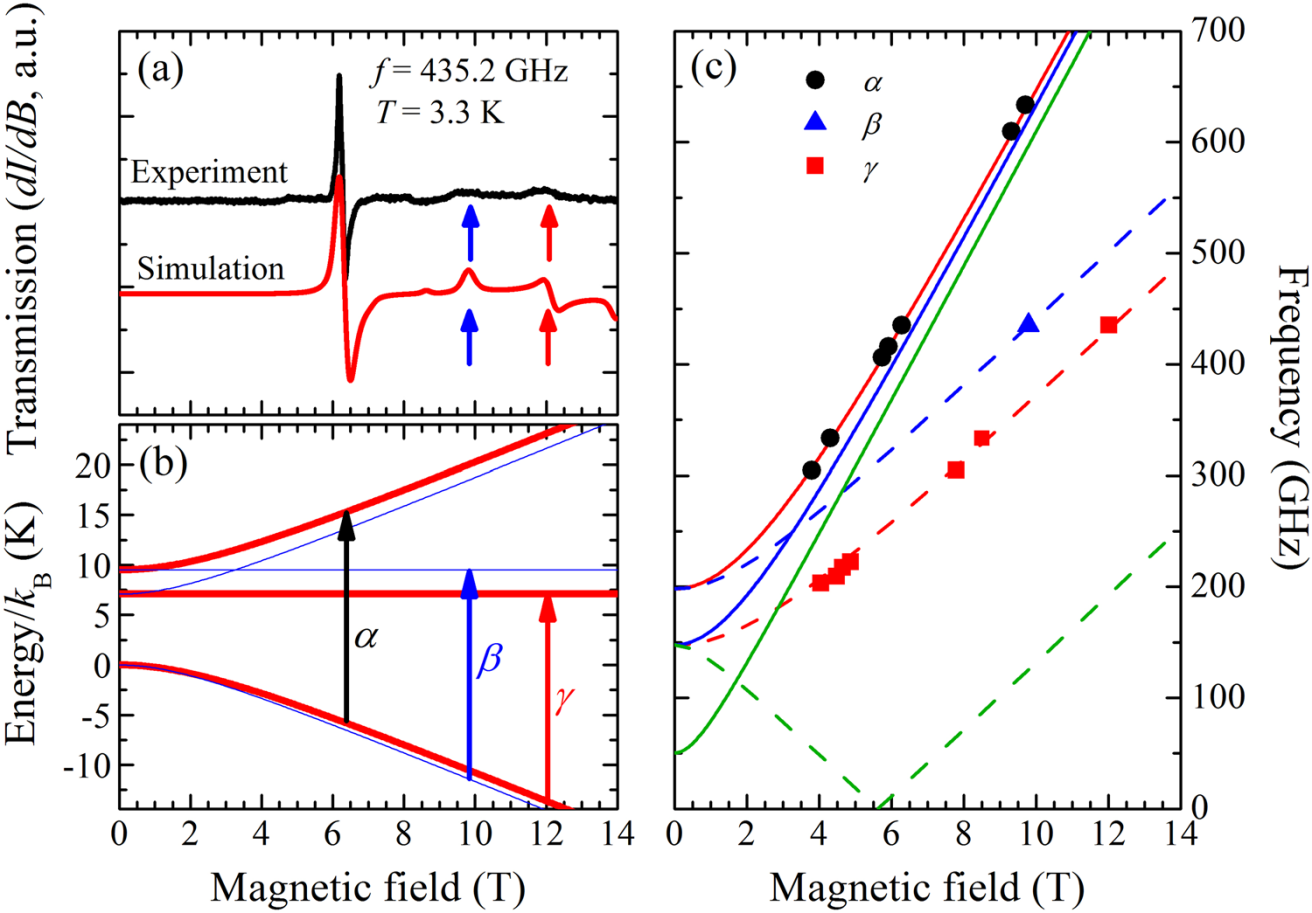
(**a**) The experimental (black) and simulated (red) ESR powder spectra at 435.2 GHz. The simulation was performed employing eq. 1 and the parameters given in the main text. (**b**) The energy level diagram with the external applied field being parallel to the *x*-axis (red thick lines) and the *y*-axis (thin blue lines). The vertical arrows correspond to ESR transitions observed at 435.2 GHz. (**c**) Simulations for frequency vs. field plot showing the ground ESR transition positions, at 3.5 K, with the field applied parallel to the *x* (red), *y* (blue) or *z* (green) axis. The points are the measured *α*, *β* and *γ* transitions while the solid lines are the simulation. The thick and dashed lines correspond to half-field transitions and normal ground state ESR transitions, respectively.

A representative experimental ESR spectrum at 3.3 K and 435.2 GHz is shown in Fig. 3a. The measured data is compared to a simulated spectrum (with *D* =  + 8.30 K, *E* = 1.21 K, *g*_xy_ = 2.20 and *g*_z_ = 2.17) as determined from the energy-level diagram for each Ni(II) site resulting from the Hamiltonian in eq. 1. The model reproduces the field positions and relative intensities of *α*, *β*, and *γ* transitions.

In this powder simulation the *β* and *γ* transitions are labeled as the ground state transitions with the field aligned parallel to the local *x*- and *y*-axis of the Ni(II) ions respectively (Fig. 3b). The *α* transition is attributed to an excitation with the field parallel to the local *x*-axis, but occurs between the two states of *m*_x_ = −1 and *m*_x_ =  + 1 with |Δ*m*_x_| = 2 where *m*_x_ is the spin projection onto its local *x*-axis. Typical ESR transitions for an applied field along *x* occur between states with |Δ*m*_x_| = 1. However, when the applied field is comparable to the zero-field splitting *D*, a strong mixing between *m*_x_ states is expected such that *m*_x_ is no longer a good quantum number and the ESR selection rule (|Δ*m*_x_| = 1) does not hold rigorously^33^.In this case the *α* transition is attributed to a half-field transition, exhibiting a large effective *g* factor (*g*_eff_ ≈ 4) that is almost twice that of a normal ESR transition (*g*_eff_ ≈ 2)^32^. Such a transition is typically observed in powder ESR spectra for pseudo-octahedral Ni(II) when the experimental frequency is comparable to *D*^34^.

If the ESR spectra are simulated instead with an easy-axis *D* = −8.30 K then the field of the *α* transition is reproduced; however, the *β* and *γ* transitions appear at much higher fields and the simulation cannot account for all transitions simultaneously. Therefore, the ESR results strongly favor the presence of an easy-plane anisotropy. The rhombic distortion term (*E*) is also essential to explain the ESR data since the separation between the *β* and *γ* transitions depend solely on *E*. When *E* = 0, *β* and *γ* morph into a single transition and the intensity of this transition would be similar to that of the *α* transition.

Comparing the observed peak positions of the frequency-dependent ESR transitions at 3.5 K to the expected peak positions from simulated ESR spectra (Fig. 3c), a good agreement was obtained when *D* = + 8.30 K, *E* = 1.21 K, *g*_xy_ = 2.20 and *g*_z_ = 2.17. These parameters are all well constrained by the data. The simulation also reveals that at high frequencies all half-field transitions are closely spaced in field and move slowly apart as frequency decreases. This may account for the observation that the transition labelled *α* becomes significantly broader at low frequency, exhibiting a complex fine structure for *ν* < 400 GHz (Fig. 2b).

### Pulsed-field magnetization

**Figure 4:**
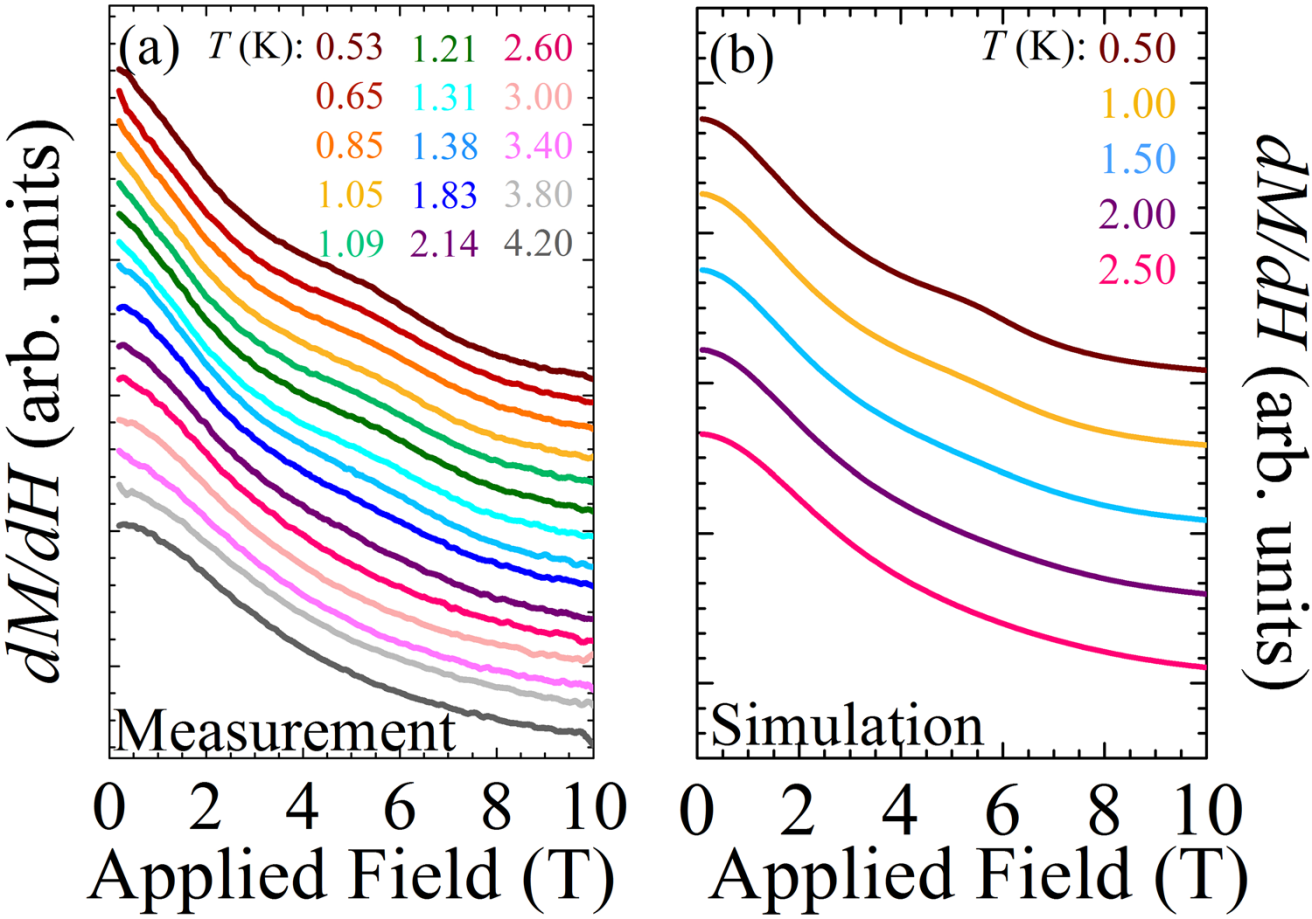
(**a**) *dM/dH* vs. applied field measured for a powdered sample with pulsed-field magnetization for 0.53 ≤ *T* ≤ 4.20 K. A bump emerges at a field of 5–6 T, and whilst the applied field of the feature is temperature independent, its magnitude decreases as the temperature increases. (**b**) The simulated *dM*/*dH* reproduces the kink between 5–6 T, which becomes less pronounced on warming. (Data have been offset for clarity in both panels).

A small bump in the powder differential susceptibility (d*M/*d*H)* develops at ≈ 5 T as the sample is cooled below 2 K (Fig. 4a). The field at which this feature occurs is *T* independent. Given the easy-plane anisotropy of the Ni(II) ions, eq. 1 implies that for a field applied along the local *z*-axis (i.e., the hard-axis) a transition from an *m*_*z*_ = 0 to *m*_*z*_ = 1 ground state occurs at a critical field (*H*_c_):

2 $$g{\mu }_{{\rm{B}}}{\mu }_{0}{H}_{{\rm{c}}}=\sqrt{{D}^{2}-{E}^{2}}.$$

At *T* = 0, this level crossing induces a sharp step in the *z*-component of the magnetization (*M*_*z*_) resulting in a large peak in d*M*_*z*_/d*H*. For *T* > 0, this step-like feature is thermally broadened due to occupation of energy levels above the ground state. If *H* is applied within the easy-plane the ground state does not cross any other spin-states (Fig. 3b) so *M*_*x*,*y*_ will resemble a Brillouin function, smoothly approaching saturation as the separation of energy levels becomes larger than *k*_B_*T*. Typical experimental powder *M*(*H*) curves are shown in Fig. S3 of the SI.

A simulation of the powdered differential susceptibility was performed by taking the *D*, *E*, *g*_*z*_ and *g*_*xy*_ values deduced from ESR, the eigenvalues of eq. 1 for various orientations of the field and deriving the magnetization from the resultant eigenvalues using statistical mechanics. A small bump in the simulated d*M*/d*H* (Fig. 4b) is reproduced at low *T*s compared to the ZFS. This feature results from the level crossing for fields parallel to *z* and has small amplitude primarily due to the effects of powder averaging. Since the level crossing giving rise to the bump in d*M*/d*H* is only expected when *D* > 0, this behavior unambiguously points to easy-plane anisotropy in [H_2_F]_2_[Ni_3_F_6_(Fpy)_12_][SbF_6_]_2_.

### Heat capacity

**Figure 5:**
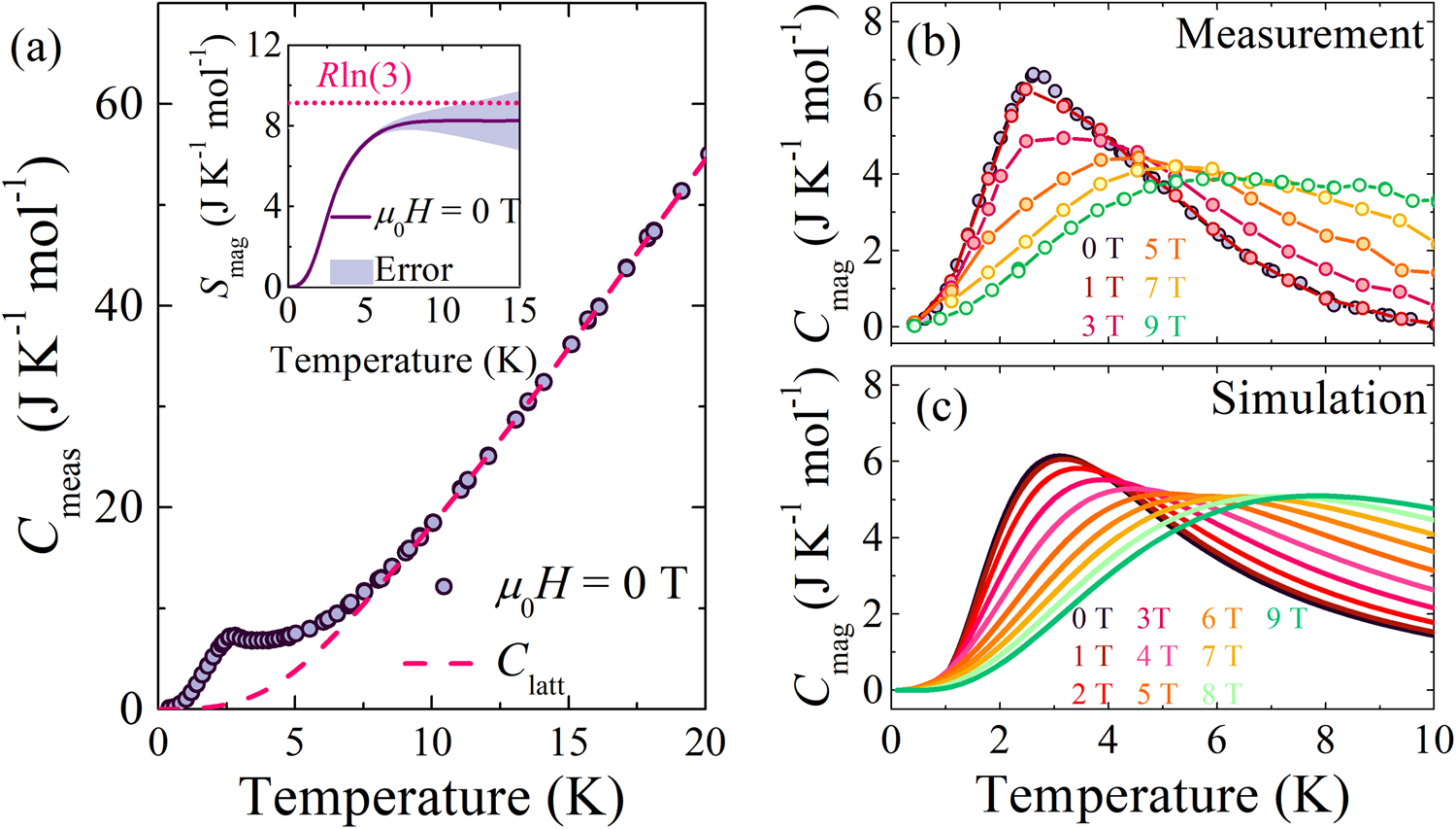
(**a**) Main Panel: Measured heat capacity (*C*_meas_) vs. temperature (*T*) of polycrystalline [H_2_F]_2_[Ni_3_F_6_(Fpy)_12_][SbF_6_]_2_, compared to the lattice contribution (*C*_latt_). Inset: The entropy change deduced by integrating the magnetic contribution to the heat capacity *C*_mag_ is compared to *R*ln3 (dotted line). The shaded region estimates the error on the calculated entropy from the lattice subtraction. (**b**) Magnetic heat capacity vs. temperature for polycrystalline [H_2_F]_2_[Ni_3_F_6_(Fpy)_12_][SbF_6_]_2_. (**c**) Simulated magnetic heat capacity vs. temperature for applied fields 0 ≤ *μ*_0_*H* ≤ 9 T.

The zero-field powder heat capacity *C*_meas_ (Fig. 5a) displays a broad maximum at 2.5 K superimposed on a gradually rising background due to the lattice contribution, *C*_latt_. To approximate *C*_latt_, the data for *T* > 10 K were modelled (see SI) with one Debye and one Einstein phonon mode (solid red line). The spin entropy, *S*_mag_(*T*), was then calculated from the magnetic heat capacity, *C*_mag_ = *C*_meas_ − *C*_latt_, using:

3 $${S}_{{\rm{mag}}}(T)={\int }_{0}^{T}\frac{{C}_{{\rm{mag}}}(T^{\prime} )}{T^{\prime} }{\rm{d}}{T}{^{\prime} }\,,$$

where *C*_mag_ = 0 at *T* = 0 is assumed. *S*_mag_ approaches a high-*T* value of 8.3 ± 1.3 J/mol·K (Fig. 5a, inset), indicating the spin-entropy change associated with cooling through the broad maximum in *C*_meas_ is consistent with *R*ln3. This is the full value expected for *S* = 1 moments cooled from a paramagnetic state to a single ground state, thus, the broad maximum is attributed to a Schottky anomaly arising from the easy-plane zero-field splitting of Ni(II) spin-states. The peak position in *C*_mag_ shifts to higher *T*s and is reduced in height as the field is increased (Fig. 5b), which arises from the increased splitting of energy levels in an applied field.

**Figure 6:**
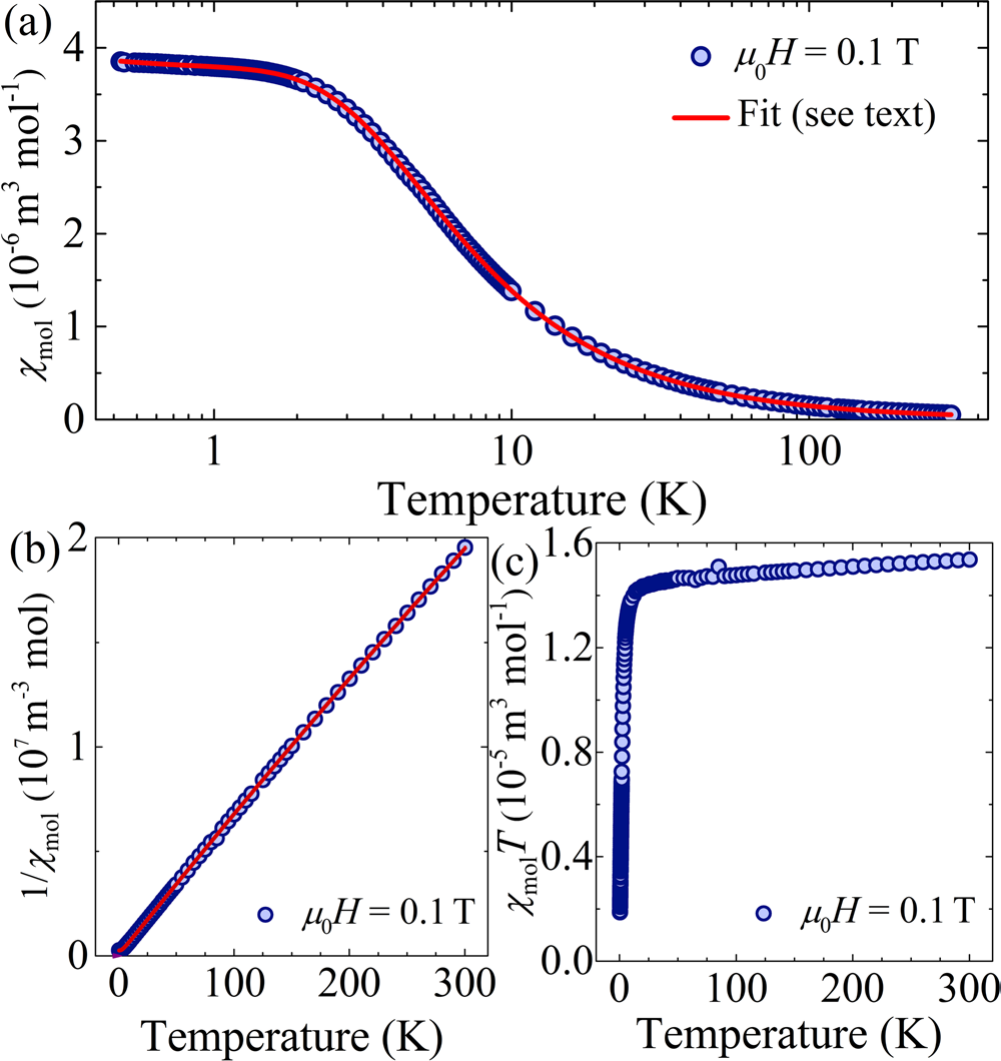
(**a**) Measured molar susceptibility (*χ*_mol_) vs. temperature (*T*) for a powdered sample (points) fitted to eq. 7 (line). Fit parameters are given in the text. (**b**) 1/*χ*_mol_ vs. *T* and the inverse of the fit from panel (**a**) is plotted for comparison. (**c**) *χ*_mol_*T* vs. *T* indicates a small temperature independent paramagnetic component to the measurement, evidenced as a positive gradient at room temperature. The significant decrease in the product *χ*_mol_*T* below approximately 15 K is indicative of the onset of single-ion anisotropy at low temperatures in the powdered sample.

The powder magnetic heat capacity was modeled with the eigenvalues of eq. 1 for various orientations of the field, using the *D*, *E*, *g*_xy_ and *g*_z_ values deduced from ESR. The simulated form of *C*_mag_ (Fig. 6c) reproduces several features of the measured data. A peak in the simulation moves to higher *T* as the field increases as a result of the aforementioned Zeeman splitting of energy levels. The simulated peak height is suppressed in fields up to ≈5 T and, thereafter, the change with increasing field is much smaller. This trend in peak height is very similar to that observed in the measured *C*_mag_, while the field dependence of the *T* of the peak in *C*_mag_ is also well described by the calculation (see Fig. S4).

### Magnetic susceptibility

The powder molar susceptibility measured in a 0.1 T applied field, *χ*_mol_ (Fig. 6, points), increases upon cooling and begins to plateau below ≈2 K, as expected for independent Ni(II) ions with finite ZFS^29^. By considering fields along the *i* = *x*, *y*, and *z* directions in eq. 1, the susceptibility components,* χ*_*i*_, are determined to be

4 $${\chi }_{x}=\frac{2{\mu }_{0}{N}_{{\rm{A}}}{\mu }_{{\rm{B}}}^{2}{g}_{{xy}}^{2}}{D+E}\frac{1\,-{{\rm{e}}}^{-{\beta }({D}+E)}}{1\,+\,2{{\rm{e}}}^{-{\beta }D}\,\cosh ({\beta }E)},$$

5 $${\chi }_{y}=\frac{2{\mu }_{0}{N}_{{\rm{A}}}{\mu }_{{\rm{B}}}^{2}{g}_{xy}^{2}}{D-E}\frac{1\,-\,{{\rm{e}}}^{-{\beta }(D+E)}}{1+2{{\rm{e}}}^{-{\beta }D}\,\cosh ({\beta }E)}\,$$

and

6 $${\chi }_{z}=\frac{2{\mu }_{0}{N}_{{\rm{A}}}{\mu }_{{\rm{B}}}^{2}{g}_{z}^{2}}{E}\frac{{e}^{-{\beta }D}\,\sinh ({\beta }E)}{1+2{e}^{-{\beta }D}\,\cosh ({\beta }E)},$$

where *β* = 1/*k*_B_*T*. The measured data were fitted over the full *T* range to the powder-average model

7 $${\chi }_{{\rm{mol}}}=({\rm{1}}-{\rm{\rho }})(\frac{{\chi }_{x}{+{\rm{\chi }}}_{y}\,+\,{\chi }_{z}}{3})+{\rho }{\chi }_{{\rm{para}}}+{\chi }_{0},$$

where a paramagnetic impurity term *χ*_para_ accounts for a slight increase in *χ*_mol_ on cooling below 2 K and a temperature independent paramagnetic term, *χ*_0_, models the linear response of *χ*_mol_(*T*)*T* at high *T*s (Fig. 6c). Assigning a *g*-factor of 2.2 to a *S* = 1 impurity phase and approximating *g*_*xy*_ ≈ *g*_z_ = *g*, the fitted parameters are: *g* = 2.14(1), *D* = +8.05(1) K, *E* = 1.73(3) K, *ρ* = 0.42(1)% and *χ*_0_ = 3.2(6) × 10^−9^ m^3^mol^−1^. The model (Fig. 6a and b, line) accounts for the measured data over the full *T*-range measured; the concentration of paramagnetic impurity is small, the *g*-factor agrees with the powder average ESR value to within 5% while *χ*_0_ is comparable to that observed in similar octahedral Ni(II) complexes^35^ and accurately models the linear behavior of *χ*_mol_(*T*)*T* at high *T*s (Fig. 6c).

Additional susceptibility studies using a 0.01 T dc magnetic field yielded no additional features, while ac susceptibility measurements below 10 K revealed no out-of-phase component down to 2 K for the ac frequencies in the range 15 ≤ *f* ≤ 1500 Hz and in applied dc fields of 0 and 0.1 T (see Fig. S5 in the SI). These measurements confirm both the weak nature of the Ni(II) spin exchange interactions and that the dominant anisotropy in this temperature range is easy-plane in nature: an easy-axis anisotropy would give rise to a slow-relaxation of the magnetization.

### Exchange interactions and DFT

The unexpectedly weak nature of the Ni-Ni spin-exchange interactions were investigated by considering two alternative coupling scenarios based on Ni(II) ions arranged on a triangular array as realized in the rhombohedral unit cell of [H_2_F]_2_[Ni_3_F_6_(Fpy)_12_][SbF_6_]_2_. Accurate energies^36^ for these candidate structures and their magnetic configurations were calculated using density-functional theory (DFT) as implemented in the CASTEP package^37^ with PBE pseudo-potentials^38^. Further details of the DFT calculations are described in the SI (see also Figs S6–S9).

**Figure 7:**
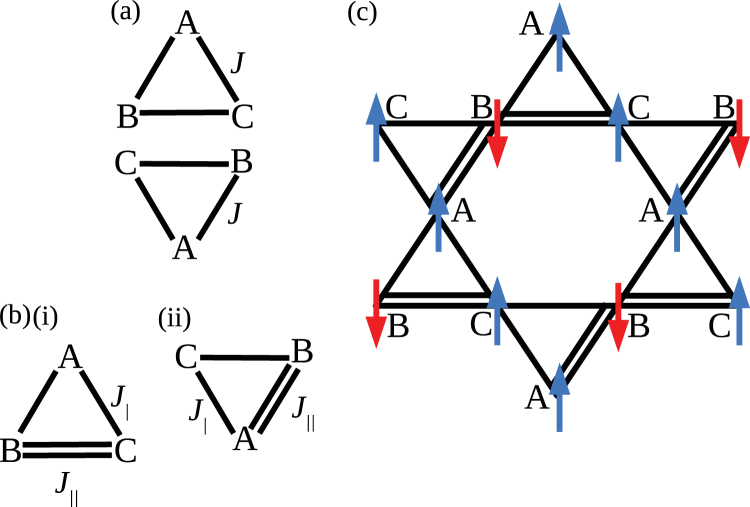
Orientations of Ni-triangles (within the rhombohedral unit cell) shown for (**a**) the 3-link model, where all triangles are identically linked and (**b**) the two-link model. Ni(II) ions are labeled A, B, and C. The kagome structure is formed by joining vertices in one triangle with their identically labeled vertices in the other, as has been done for the 2-link case in (**c**). In (**b**) exchange pathways between linked (unlinked) Ni(II) ions are represented by double (single) lines with the exchange constant *J*_||_ (*J*_|_). (**c**) Lowest energy configuration for a magnetic model of Ising-like spins for the 2-link model.

We first consider an idealized configuration whereby three equivalent Ni ions interact (we call this the ‘3-link’ model; Fig. 7a). The model, which is not intended to be realistic, does not involve any structural disorder, but provides a useful comparison. It comprises triangles where all three Ni(II) ions are connected equivalently through an intact H_3_F_4_^−^ moiety. The central F-ion of the moiety in each triangle is situated close to the plane formed by the triangle, and all exchange pathways have the same exchange constant *J*. Since all triangles are equivalent, these tile to give the usual kagome lattice. Including spin degrees-of-freedom we find that the ground state for this configuration is FM.

The second model is intended to simulate the disorder revealed by the X-ray results. In this model, two Ni ions in a triangle strongly interact owing to superexchange via a connecting H_2_F_3_^−^ moiety with the other Ni ion only weakly interacting (we refer to this as a ‘2-link’ geometry; Fig. 7b). This forms a triangle with one bond expected to provide a relatively strong interaction (with exchange constant *J*_||_) and two that are weaker in comparison (with exchange constant *J*_|_). Since the simulation of true disorder is difficult in DFT, we opt for a compromise approach in which calculations were performed on different ordered configurations of these triangles, since such triangles would likely be the building blocks of the disordered structure. Several relative orientations of two of these triangles, along with different relative positions of the connecting moiety, have been examined in order to find the most energetically stable. We find that the lowest energy configuration is formed from the triangles shown in Fig. 7b, where the central F-ion is displaced out of the plane in (i) and remains roughly in-plane in (ii). When tiled these give the configuration shown in Fig. 7c. We note that the next-lowest energy structural configuration for this material was found to occur 1220 K higher in energy than the lowest energy configuration described above. Examination of the energies of different spin states for this configuration led us to predict an antiferromagnetic (AFM) (or possibly ferrimagnetic) structure, part of which is shown in Fig. 7c. This comprises alternating stripes of up and down spins which form parallel to the direction in which nearest-neighbor Ni(II) ions are weakly connected, with spins that are strongly connected always AFM aligned. Note that since we use collinear (Ising) spins in these calculations, this magnetic structure is likely to approximate the true ground state, but its stability relative to the ferromagnetic (FM) state (which lies ≈ 6.3 K higher in energy) is sufficient to indicate a degree of antiferromagnetism in the 2-link structure.

By comparing the energies of the magnetic configurations we may calculate the Heisenberg exchange constants for each model. Using the simplified Hamiltonian, ${\mathcal H}$ = Σ_*i*,*i*+*â*_*J*_*i*,*i*+*â*_*S*_*i*_ · *S*_*i*,*i*+*â*_, where *i* denotes a site and *â* the hopping vector to a nearest-neighbor, we find that the 3-link geometry has a FM exchange constant *J* = −8 K along every nearest-neighbor exchange pathway, while the 2-link geometry has AFM exchange *J*_||_ = 0.4 K along the strongly interacting bonds and *J*_|_ = 0.2 K along the weak ones. Note that the precise values of the exchange constants are subject to a systematic uncertainty of order ~0.1 K owing to the limit of what present exchange functionals used in DFT calculations can reliably calculate in this case, so the precise values for the 2-link geometry should be treated with caution. Despite this, it is clear that the value of the 3-link exchange constant is significantly larger than is observed experimentally, while the 2-link exchange constants are much smaller and thus consistent with the measured data.

Although we believe the system to possess a *XY-*like spin anisotropy, the Ising-like magnetic model considered here allows us to evaluate the magnetic exchange energy scales at work in the system. Our approach involves determining the energy differences between ordered spin configurations that differ by a number of reversed spins. The value of the magnetic exchange derived therefore assumes that nearest neighbor spins obey *S*_*i*_·*S*_*i*,*i*+*â*_ =  ± 1. If this is not the case, then the error in this assumption is absorbed into the value of *J* that is derived. Indeed, the single-ion parameters extracted from the ESR and magnetometry data indicate that, because of a relatively strong easy-plane anisotropy and a staggering of the octahedra between adjacent nickel ions, the spins will not be collinear at low temperatures. In any case, we believe the model provides a valuable insight into the relative energy scales that determine the magnetism of the compound.

Spin density plots (see Fig. S10) suggest an explanation as to why *J* is much larger than *J*_||_ and *J*_|_. In the FM spin configuration of the 3-link structure, we find delocalized spin on the central F ion that links the Ni(II) ions, suggesting that overlap between spins on different Ni(II) ions stabilizes this state. However, for an AFM configuration based on this model, any delocalization on the F-ion is negligible, suggesting that much less overlap between spins is taking place. The energy cost of flipping Ni-spins is then large leading to the large *J*-value. For the 2-link geometry we discover that there is some spin delocalization along the strong bond in both the lowest-energy AFM and the higher-lying FM configurations. This implies that the energy required to flip a spin is less than in the 3-link case. In the case of the weak bonds, there is very little delocalization between spins for either configuration, and so spins on neighboring sites have little influence over each other. In that case, very little energy is required to reverse the orientation of a spin.

In summary, the DFT calculations indicate that the preferred structure of this material is such that one exchange pathway in each spin-triangle is structurally distinct from the other two and that the resulting spin-density gives rise to much reduced exchange energies between spins, compared to the case in which all pathways are identical. This result then explains the experimental observation that the effective exchange *J* in this material must be significantly lower than those measured in related Ni(II) coordination compounds. We attribute the reduced *J* in [H_2_F]_2_[Ni_3_F_6_(Fpy)_12_][SbF_6_]_2_ to the distribution of disordered exchange paths, as determined from our 100 K X-ray measurements, though in all probability, persisting to even lower temperatures. The low value of the exchange constant explains why no long-range AFM order is observed in the muon-spin relaxation experiments

## Conclusions

[H_2_F]_2_[Ni_3_F_6_(Fpy)_12_][SbF_6_]_2_ contains pseudo-octahedral Ni(II) ions that occupy the vertices of a 2D kagome lattice. Bond disorder among intervening H_2_F^+^ moieties reduces the superexchange interaction between Ni(II) sites as revealed by thermodynamic probes and DFT. While the exchange couplings between spins were predicted to be significant (≈ 8 K) the surprising revelation was that a relatively small distortion along one of the three possible exchange pathways dramatically reduced the interaction strength (to ≤ 1 K), a result corroborated by our DFT calculations and confirmed via X-ray diffraction and magnetometry measurements. While this distortion appears to relieve any signs of magnetic frustration, the inherently small exchange energies and structural disorder prevent the onset of long-range ordering at the lowest temperatures measured.

The significance of this work demonstrates the clear impact of bond disorder on the magnetic properties of a potentially frustrated *S* = 1 system. Our materials design methodology based on strong H···F bonds continues to provide novel architectures and model systems in which to test theories of quantum matter. Specifically, we suggest that it may be possible to create a similar lattice that promotes those large exchange energies associated with the 3-link model, allowing the full effect of geometric frustration to be studied in a polymeric system. We are exploring other synthetic routes with the aim of creating a fully frustrated kagome lattice assembled from strong hydrogen bonds.

## Methods

### Synthesis

All reagents were obtained from commercial sources and used as received. Plasticware was utilized in all chemical manipulations and samples stored in plastic vials. While stirring, hexafluoroantimonic acid (60% aqueous solution) was slowly added to a suspension of NiCO_3_ until a pale green solution was produced and all CO_2_ liberated. The solvent was removed under dynamic vacuum to produce moist Ni(SbF_6_)_2_. In a typical synthesis of [H_2_F]_2_[Ni_3_F_6_(Fpy)_12_][SbF_6_]_2_, Ni(SbF_6_)_2_·yH_2_O (0.1559 g, 0.29 mmol; based on anhydrous formula weight) was dissolved in 5-mL of 3-fluoropyridine. In a separate beaker, NH_4_HF_2_ (0.0169 g, 0.30 mmol) was dissolved in 1-mL of H_2_O. The two solutions were slowly mixed to give a pale blue-green mixture. Upon slow solvent evaporation, blue blocks of the product were obtained in high yield. The infrared spectrum of neat [H_2_F]_2_[Ni_3_F_6_(Fpy)_12_][SbF_6_]_2_ is shown in Fig. S1.

### X-ray structure determination

A pale blue block of [H_2_F]_2_[Ni_3_F_6_(Fpy)_12_][SbF_6_]_2_ measuring 0.355 × 0.237 × 0.146 mm was selected and used for the X-ray crystallographic analysis. The X-ray intensity data were measured at 100 K using a Bruker APEX II X-ray diffractometer equipped with a TRIUMF curved-crystal monochromator (*λ* = 0.71073 Å). The unit cell, space group and data integration were performed with the Bruker APEX II and SAINT software package^39^. Structure solution and refinement were carried out using SHELXT-2914 and SHELXL-2014/7, respectively^40,41^. Data were corrected for absorption effects using the multi-scan method (SADABS)^42^. Crystallographic refinement details are given in Table 1 while selected bond lengths (Å) and bond angles (°) are listed in Table 2.

**Table 2:** Selected bond lengths (Å) and bond angles (°) for [H_2_F]_2_[Ni_3_F_6_(Fpy)_12_][SbF_6_]_2_.

Ni(1)-F(3)	2.000(1)	N(1)-Ni(1)-N(1)	180
Ni(1)-N(1)	2.145(1)	N(1)-Ni(1)-N(2)	91.22(5)
Ni(1)-N(2)	2.101(1)	N(1)-Ni(1)-F(3)	91.56(5)
H(1C)-F(3)	1.392	N(2)-Ni(1)-F(3)	88.97(5)
H(2C)-F(3)	1.444	Ni(1)-F(3)-H(1C)	144.45
H(1C)-F(1C)	0.96	Ni(1)-F(3)-H(2C)	153.49
H(2C)-F(2C)	0.96	C(1)-N(1)-C(5)	117.2(2)
F(1C)···F(3)	2.352(1)	F(1)-C(1)-C(2)	118.5(2)
F(2C)···F(3)	2.404(2)	F(1A)-Sb(1A)-F(2A)	91.4(5)
N(1)-C(1)	1.334(2)	F(1A)-Sb(1A)-F(3A)	90.4(5)
N(1)-C(5)	1.333(2)	Ni···Ni (intraplane)	7.181(1)
F(1)-C(2)	1.348(3)	Ni···Ni (interplane)	11.105(1)

### DC field magnetization measurements

The magnetic moment (*M*) was measured in an applied field of *μ*_0_*H* = 0.1 T for temperatures in the range (i) 1.8 ≤ *T* ≤ 300 K, with a Quantum Design 7-T SQUID magnetometer; and (ii) 0.48 ≤ *T* ≤ 2 K with an *i*Quantum ^3^He insert to the SQUID magnetometer. The discrepancy between the two measurements at the same temperature was less than 3%. In the linear limit, the molar magnetic susceptibility (χ_mol_) was deduced from these measurement using χ_mol_ = *M*/*nH*, where *n* is the number of moles of sample.

### Pulsed-field magnetization studies

*M*(*H*) measurements were conducted at the Nicholas Kurti Magnetic Field Laboratory, University of Oxford (UK). A pulse of maximum field *μ*_0_*H* = 10 T, and rise-time to full field of ≈5 ms was used to measure the sample moment for temperatures in the range 0.53 ≤ *T* ≤ 4.55 K. Details of the technique can be found in ref.^43^. The data shown in Fig. 4 have been interpolated and smoothed using a Savitzsky-Golay method.

### Electron-spin resonance

High-frequency ESR measurements were performed on powdered samples using the Homodyne 15-T transmission ESR spectrometer at the National High Magnetic Field Laboratory, Tallahassee, FL (USA). The sample was finely ground and restrained by KBr. A ^4^He flow cryostat was used for temperature control.

### Heat capacity

The low-*T* heat capacity was measured between 0.4 ≤ *T* ≤ 20 K for applied fields 0 ≤ *μ*_0_*H* ≤ 9 T with a ^3^He insert to a Quantum Design PPMS, using a 1.82(5) mg polycrystalline sample mounted on a sapphire stage using Apiezon-N grease. The heat capacity data were corrected for the (field-dependent) background of the stage/grease by subtracting the heat capacity measured with no sample in place. The data in Fig. 5(b) are the result of averaging over repeat measurements.

### Muon-spin relaxation

Zero-field *μ*^+^SR measurements were performed on powder samples using the Low Temperature Facility (LTF) and General Purpose Surface-Muon (GPS) spectrometers at the Swiss Muon Source (S*μ*S), Paul Scherrer Institut, Switzerland. The samples were packaged in Ag foil envelopes (foil thickness 12.5 *μ*m) and mounted either on a silver plate (LTF) or taped to a silver fork (GPS).

### Simulations

Numerical calculations of the expected heat capacity (*C*_p_) and magnetization (*M*) of a powdered sample were performed using MATLAB. For a field applied in a particular direction with respect to the local *z*-axis of each Ni(II) ion, the calculation of the eigenvalues of the Hamiltonian in eq. 1 uses a routine within MATLAB. The three resultant eigenvalues can be used to derive the *C*_p_ and *M* from the partition function (see e.g. ref.^44^). By averaging these quantities over 400 different orientations of the field, the expected *C*_p_ and *M* of a powdered sample could be estimated.

### Data Availability

CCDC 1824114 contains the supplementary crystallographic data for this paper. These data can be obtained free of charge via www.ccdc.cam.ac.uk/data_request/cif, or by emailing data_request@ccdc.cam.ac.uk, or by contacting The Cambridge Crystallographic Data Centre, 12 Union Road, Cambridge CB2 1EZ, UK; fax: +44 1223 336033. Data presented in this paper resulting from the UK effort will be made available at http://wrap.warwick.ac.uk/99677.

## Electronic supplementary material

Supplementary Information
