# Supplementary material for: Implications of bond disorder in a S=1 kagome lattice
Source: Sci Rep. 2018 Mar 16;8:4745. doi: 10.1038/s41598-018-23054-6 (PMC5856763; doi:10.1038/s41598-018-23054-6)
Supplement: Supplementary file 1 — Supplementary Information [file 41598_2018_23054_MOESM1_ESM.pdf]

Supplementary Information for:

Implications of bond disorder in a $S = 1$ kagome lattice

Jamie L. Manson,[1*] Jamie Brambleby,[2] Paul A. Goddard,[2] Peter M. Spurgeon,[1] Jacqueline A. Villa,[1] Junjie Liu,[3] Saman Ghannadzadeh,[3] F. Foronda,[3] John Singleton,[4] Tom Lancaster,[5] Stewart J. Clark,[5] Iorwerth O. Thomas,[5] Fan Xiao,[5] Robert C. Williams,[5] Francis L. Pratt,[6] Stephen J. Blundell,[3] Craig V. Topping,[3] Christopher Baines,[7] Charles Campana,[8] and Bruce Noll[8]

[1]*Department of Chemistry and Biochemistry, Eastern Washington University, Cheney, WA 99004 United States*

[2]*Department of Physics, University of Warwick, Coventry CV4 7AL United Kingdom*

[3]*Clarendon Laboratory, Department of Physics, University of Oxford, Oxford OX1 3PU United Kingdom*

[4]*National High Magnetic Field Laboratory, Los Alamos National Laboratory, Los Alamos, NM 87545 United States*

[5]*Center for Materials Physics, Durham University, Durham DH1 3LE United Kingdom*

[6]*STFC, ISIS Pulsed Muon Facility, Rutherford-Appleton Laboratory, Chilton, Oxfordshire OX11 0QX United Kingdom*

[7]*Paul Scherrer Institut, Laboratory for Muon-Spin Spectroscopy, CH-5232 Villigen PSI, Switzerland*

[8]*Bruker AXS, Inc., Madison, WI 53711 United States*

RECEIVED DATE (automatically inserted by publisher); jmanson@ewu.edu

1. Muon-spin relaxation experiments.

In a muon-spin rotation (μ^+SR) experiment, spin-polarised μ^+ ions are implanted in a sample such that the muons occupy interstitial positions in the crystal lattice.[1] The population of muons then decreases on the time-scale set by their 2.2 μs mean-lifetime. By-products of the muon-decay are positrons, which are preferentially emitted parallel to the instantaneous direction of the μ^+ polarisation at the decay event. Two scintillators, positioned in the forward (F) and backward (B) positions relative to the μ^+-beam direction, record the number of positrons [N_i(t), i = F, B] as a function of time, t, during an experiment. The asymmetry, $A(t)$, parameterises the preferred direction of positron emission via

$$A(t) = \frac{N_{\mathrm{F}}(t) - \alpha N_{\mathrm{B}}(t)}{N_{\mathrm{F}} + \alpha N_{\mathrm{B}}(t)}, \qquad \text{(S1)}$$

where α is an experimentally determined constant accounting for differences in the detectors. The asymmetry is proportional to the muon polarization along the beam direction.

Example asymmetry spectra for $[H_2F]_2[Ni_3F_6(Fpy)_{12}][SbF_6]_2$, at several temperatures are shown in Fig. S1 (left). Little change is observed in the spectra between 0.019 and 10 K. In all cases, the signal shows a fast decay at early times and then relaxes roughly as an exponential function. An additional low-amplitude, low-frequency oscillation is super-imposed on the relaxing signal.

The fast relaxing component is likely due to paramagnetic muon sites and has been observed in many molecular magnets.[2] The slow exponential decay (with decay rate ~0.7 MHz) arises from the fluctuation of the electronic moments and is therefore sensitive to magnetism in the material. The weak oscillatory signal is commonly seen in fluorine compounds, indicative of F-μ^+ entangled states,[3] where the muon is strongly coupled to a spin-1/2 ^{19}F nucleus and the oscillatory frequency $\omega/2\pi$ is related to the F-μ^+ separation.

The asymmetry can therefore be best fitted to a function with several relaxing components:

$$A(t) = A_{\mathrm{fast}} e^{-\lambda_{\mathrm{fast}} t} + A_{\mathrm{slow}} e^{-\lambda_{\mathrm{slow}} t} + A_{\mathrm{F}\mu} D_z(\omega, t) e^{-\lambda_{\mathrm{F}\mu} t} + A_{\mathrm{bg}}, \qquad \text{(S2)}$$

where A_{fast} and A_{slow} are the amplitudes of the fast and slow relaxing components with relaxation rates λ_{fast} and λ_{slow}, respectively; $A_{\mathrm{F}\mu}$ and $\lambda_{\mathrm{F}\mu}$ are the amplitude and relaxation rate of the F-μ^+ signal and $D_z(\omega, t)$ is the F-μ^+ polarization function.[3] The amplitude A_{bg} accounts for the non-relaxing contribution from the muons that stop within the sample holder and cryostat tail.

The extracted ω values and the slow relaxation rate λ_{slow} are plotted against temperature in Fig. S1(a) and (b). No significant features were observed throughout the measured temperature window, notably near 2.5 K where a peak was revealed by heat capacity measurements. The average value of $\omega/2\pi$ is 1.74 MHz, corresponding to a F-μ^+ separation of 1.09 Å within the unit

cell. The lack of change in the relaxation rate λ_{slow}, which is proportional to the width of the local field distribution and the correlation time via $\lambda \propto \langle (B - \langle B\rangle)^2\rangle\tau$,[4] suggests that no significant change in static or dynamic magnetic properties is detected using muons in this material from 0.019 to 10 K. This suggests that in terms of the Hamiltonian involving interactions between Ni(II) ions will be small, which implies that for temperatures $T \geq 0.019$ K, the magnetic behavior of the sample can be largely determined from the single-ion properties.

2. Heat Capacity experiments.

The lattice contribution (C_{latt}) to the measured heat capacity of $[H_2F]_2[Ni_3F_6(Fpy)_{12}][SbF_6]_2$ (Cp) [Fig. 6(a), main text] was determined by modelling (see e.g. Ref. 5) the experimental data for $T \geq 10$ K as C_p/T vs. T with one Debye (D) and three Einstein (E) mode using:

$$C_{\mathrm{latt}} = A_{\mathrm{D}} \frac{3}{x_{\mathrm{D}}^2} \int_0^{x_{\mathrm{D}}} \frac{x^4 e^x}{(e^x - 1)} \mathrm{d}x + \sum_{i=1,2,3} A_{\mathrm{E}_i} \frac{\theta_{\mathrm{E}_i}^2}{T^2} \frac{e^{\theta_{\mathrm{E}_i}/T}}{\left(e^{\theta_{\mathrm{E}_i}/T} - 1\right)^2}, \qquad \text{(S3)}$$

where A_i and θ_i (i = D or $E_{1,2,3}$) are the characteristic amplitude and temperature of the modes and $x_D = \theta_D/T$. The resultant fitted parameters of the four modes are: A_D = 68(3) JK^{-1}mol^{-1}, θ_D = 59(1) K; A_{E1} = 112(3) JK^{-1}mol^{-1}, θ_{E1} =122(3) K; A_{E2} = 223(4) JK^{-1}mol^{-1}, θ_{E2} = 325(6) K; and A_{E3} = 370(20) JK^{-1}mol^{-1}, θ_{E3} = 1020(3) K, respectively. The model lattice heat capacity is plotted as a dashed line in Fig. 6(a) (main text).

3. Modelling of bulk thermodynamic properties of powdered samples from statistical mechanics

Magnetization. For a given field strength, H, and orientation of the field with respect the hard-axis expressed in terms of the polar angles (θ_i; ϕ_j), the eigenvalues of eq. 1 (main text) are deduced by diagonalizing the Hamiltonian. Inserting these eigenvalues into a partition function at a fixed temperature, the magnetization at a particular field strength and orientation $M(H, \theta_i, \phi_j)$, can be deduced (see e.g. Ref. 6).

The polar angles are incremented (in 20 evenly spaced steps of $\Delta\theta$ and $\Delta\phi$) such that, in total, 400 orientations of the field are considered. The average magnetization, $\langle M(H)\rangle$, at a particular applied field is approximated by:

$$\langle M(H)\rangle = \frac{\sum_{i,j} M(H, \theta_i, \phi_j) \sin\theta_i \Delta\theta\Delta\phi}{\sum_{i,j} \sin\theta_i \Delta\theta\Delta\phi}. \qquad \text{(S4)}$$

This was calculated up to μ_0H = 10 T, at $\mu_0\Delta H$ = 0.1 T intervals. By using different temperatures in the partition function for this calculation, the temperature dependence of the magnetization was also be explored (Fig. 5b, main text).

Heat Capacity. In analogous manner to calculation of the magnetization above, the heat capacity at a particular temperature and field orientation, $C_{mag}(T, \theta_i, \phi_j)$ was determined from the eigenvalues of eq. 1.[6] The direction of the applied field was incremented and C_{mag} recalculated. By considering 400 different field orientations with respect to the hard axis, the average $\langle C_{mag}(T)\rangle$ at a given field and temperature is estimated from:

$$\langle C_{\mathrm{mag}}(T)\rangle = \frac{\sum_{i,j} C_{\mathrm{mag}}(T, \theta_i, \phi_j) \sin\theta_i \Delta\theta\Delta\phi}{\sum_{i,j} \sin\theta_i \Delta\theta\Delta\phi}. \qquad \text{(S5)}$$

This calculation was performed for different fixed magnitudes of the applied field in the range $0 \leq \mu_0H \leq 9$ T, and the result is compared to the measured data in Fig. 6 (main text).

Using the D, E, g_z and g_{xy} parameters determined from ESR, the simulated heat capacity exhibits a broad maximum whose field-dependence is in good agreement with that observed in $[H_2F]_2[Ni_3F_6(Fpy)_{12}][SbF_6]_2$ (Fig. S2).

References
[1] Blundell, S. J., *Contemp. Phys.* **1999**, *40*, 175.
[2] Lancaster, T.; Blundell, S. J.; Brooks, M. L.; Baker, P. J.; Pratt, F. L.; Manson, J. L.; Landee, C. P.; Baines, C. *Phys. Rev. B* **2006**, *73*, 020410R(1)-020410R(4).
[3] Lancaster, T.; Blundell, S. J.; Baker, P. J.; Brooks, M. L.; Hayes, W.; Pratt, F. L.; Manson, J. L.; Conner, M. M.; Schlueter, J. A. *Phys. Rev. Lett.* **2007**, *99*, 267601(1)-267601(4).
[4] Hayano, R. S.; Uemura, Y. J.; Imazato, J.; Nishida, N.; Yamazaki, T.; Kubo, R. *Phys. Rev. B* **1979**, *20*, 850-859.

[5] Manson, J. L.; Schlueter, J. A.: Funk, K. A.; Southerland, H. I.; Twamley, B.; Lancaster, T.; Blundell, S. J.; Baker, P. J.; Pratt, F. L.; Singleton, J.; McDonald, R. D.; Goddard, P. A.; Sengupta, P.; Batista, C. D.; Ding, L.; Lee, C.; Whangbo, M.-H.; Franke, I.; Cox, S.; Baines, S.; Trial, D. *J. Amer. Chem. Soc*. **2009**, *131*, 6733-6747.
[6] Blundell, S. J.; Blundell, K. M. *Thermal Physics*, Oxford U. Press: Oxford, 2010.

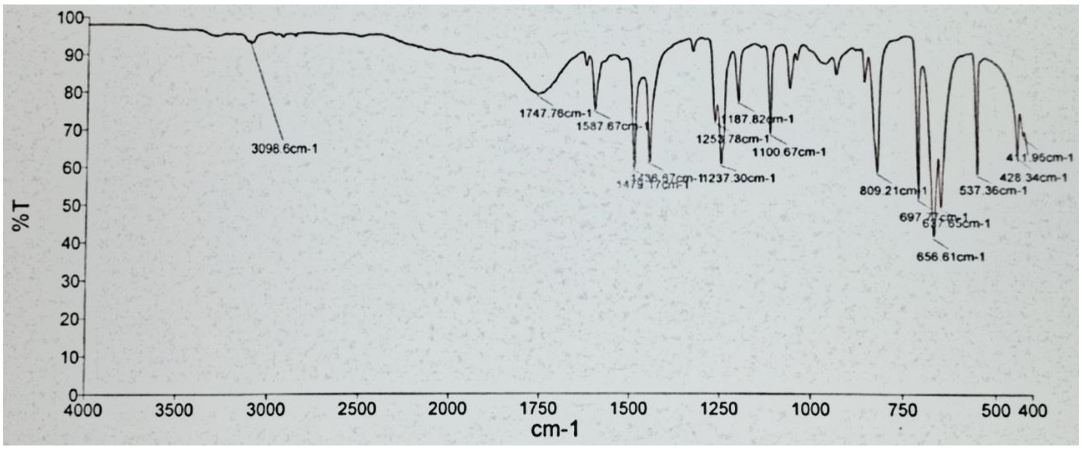

Figure S1. Room temperature IR spectrum of neat $[H_2F]_2[Ni_3F_6(Fpy)_{12}][SbF_6]_2$. The absorption at 1748 cm^{-1} is probably due to the H···F bonds in the H_2F^+ cation. The absence of O-H stretching and bending modes precludes the presence of H_2O.

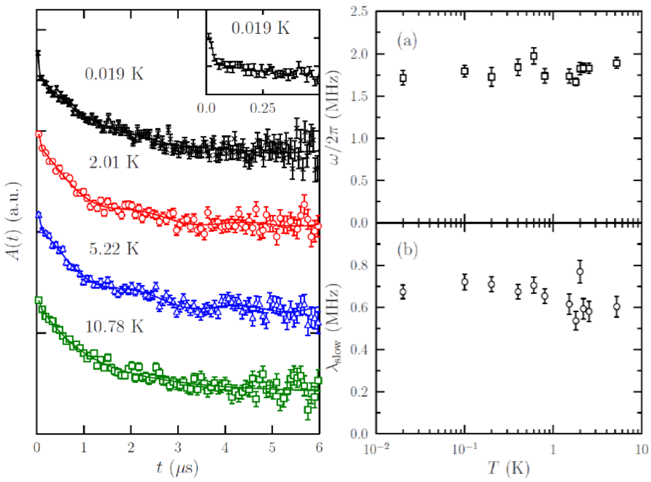

Figure S2 *Left*: Example μ^+SR spectra for $[H_2F]_2[Ni_3F_6(Fpy)_{12}][SbF_6]_2$ at selected temperatures; solid lines are fits to the data as described in the text. The inset shows the fast depolarization at early times for T = 0.019 K. *Right*: parameters resulting from fits to eqn. (S2). (a) Frequency of the F-μ^+ polarization function, (b) the slow relaxation rate λ_{slow}.

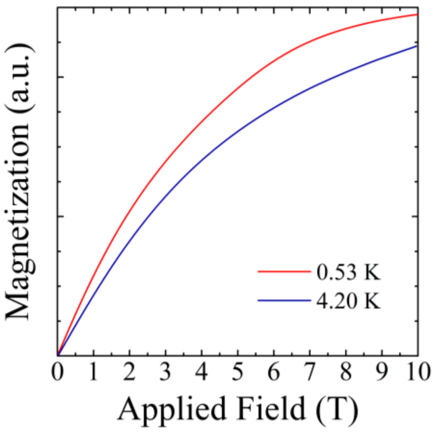

Figure S3. Pulsed-field magnetization data of polycrystalline $[H_2F]_2[Ni_3F_6(Fpy)_{12}][SbF_6]_2$ taken at two temperatures.

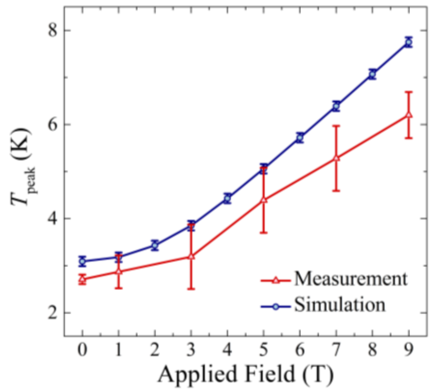

Figure S4. Simulated position of the broad maximum in the heat capacity (Fig 6c, main text) using eq.1 and the *D*, *E*, g_z and g_{xy} parameters deduced from ESR (circles). These are compared against the measured temperatures of the broad maxima in the measured data (triangles).

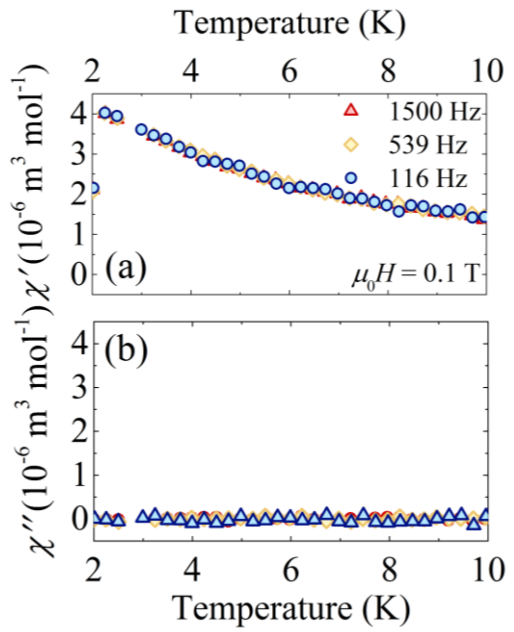

Figure S5. (a) In-phase; and (b) out-of-phase susceptibility of $[H_2F]_2[Ni_3F_6(3\text{-}Fpy)_{12}][SbF_6]_2$ recorded in a quasistatic applied magnetic field $\mu_0 H$ = 0.1 T. The ac susceptibility exhibits a negligible out-of-phase component over the temperature and frequency range of the investigation. The small value of χ' at 2 K is frequency independent, does not correspond to a rise in χ" and is not reproducible in the quasistatic linear susceptibility measurements (main text) and is therefore considered as an artifact of the measurements.

4 Density functional theory

4.1 Structural and Magnetic Models

To clarify the structure of the material and investigate the magnetic interactions between spins on different Ni ions, we consider six models for the structure of the compound Several possible orientations of the SbF_6 clusters are possible given the crystallographic data, but we have chosen a single orientation (as shown for a sample model in Fig. S6) since we are interested in the pattern of bonds between Ni ions. We will discuss the models in terms of the two triangles of Ni ions that can be seen in Figure S6, closest and furthest from the origin.

The first two models we consider have Ni ions in each triangles equivalently connected by a $H_3F_4^-$ moiety, leading to equal superexchange interactions between all spins which we label J. (We call these '3-link' models below.) As shown in Fig. S7, the difference between the two models is whether the central F ion is displaced from the plane of the triangle towards the center of the cell (called 'out-of plane' hereafter) or not (called 'in-plane'). For these 3-link models, we examine both ferromagnetic (FM) and ferrimagnetic (i.e. one spin opposite to the other two in the basic triangles) states. We expect the ferrimagnetic configurations to lead to overall antiferromagnetic (AFM) magnetic structures when the whole material is considered, so label these AFM below. The labels (A) to (C) in Fig. S7(a) show equivalent Ni ions; we can line up the ions in an in-plane triangle with their identically labeled vertices with out-of-plane triangles (and vice-versa) in order to construct a kagome lattice.

In the next four models, in-plane and out-of-plane configurations have two Ni ions linked by a $H_2F_3^-$ moiety and another not. We refer to these models as the '2-link' models. Here we have two exchange couplings, $J_{||}$ between strongly linked spin sites and $J_|$ between weakly linked sites. The in-plane and out-of-plane triangle and symmetric and asymmetric arrangements of bonds give the four possible combinations shown in Fig. S8, where $J_{||}$ couplings are labeled with double lines and the $J_|$ couplings with single lines. These models have the three spin states shown in Fig. S9. As before, the labels from (A) to (C) in Fig. S9 indicate how a kagome lattice is constructed from the triangles.

4.2 Density Functional Theory Calculation

Results for different magnetic configurations in each model were obtained using density functional theory (DFT) calculations performed on rhombohedral unit cells using CASTEP [1] version 16.1 with accurate [2] on-the-fly generated ultrasoft PBE [3] pseudopotentials using the experimentally determined crystal structure and lattice parameters. An energy convergence tolerance of 1×10^{-10} eV, a plane wave basis cutoff of 396 eV and a Grid Scaling (which fixes the standard grid size relative to the diameter of the cutoff sphere) of 1.75 were used. It was found that this converged the energy difference between the asymmetric, out-of-plane 2-link model's AFMs and AFMw magnetic configurations to within 0.01 meV. Energy calculations for the asymmetric, out-of-plane 2-link AFMs, AFMw and FM cases were repeated in order to check for consistency.

The energy differences between the 3-link models are given in Table 1(a): the FM state of the in-plane structure is found to be the most stable. Table 1(b) gives the relative separation between the lowest lying energy states of each 2-link structure; the AFMs state of the asymmetric, out-of-plane structure is the lowest-lying state.

4.3 Calculations of Exchange Constants

4.3.1 3-link asymmetric model

We begin with the Hamiltonian of the Ising-type system:

$$\hat{H} = -2J \sum_{\text{unit cells}} \left(\hat{S}_{\text{A}}\hat{S}_{\text{B}} + \hat{S}_{\text{A}}\hat{S}_{\text{C}} + \hat{S}_{\text{C}}\hat{S}_{\text{B}} \right). \tag{1}$$

The eigenvalues of the spin operator $\hat{S}$ in this case are -1, 0 and 1. From these we can calculate the energies of the AFM and FM states:

$$E_{\text{FM}} = -6J \tag{2}$$

$$E_{\text{AFM}} = 2J \tag{3}$$

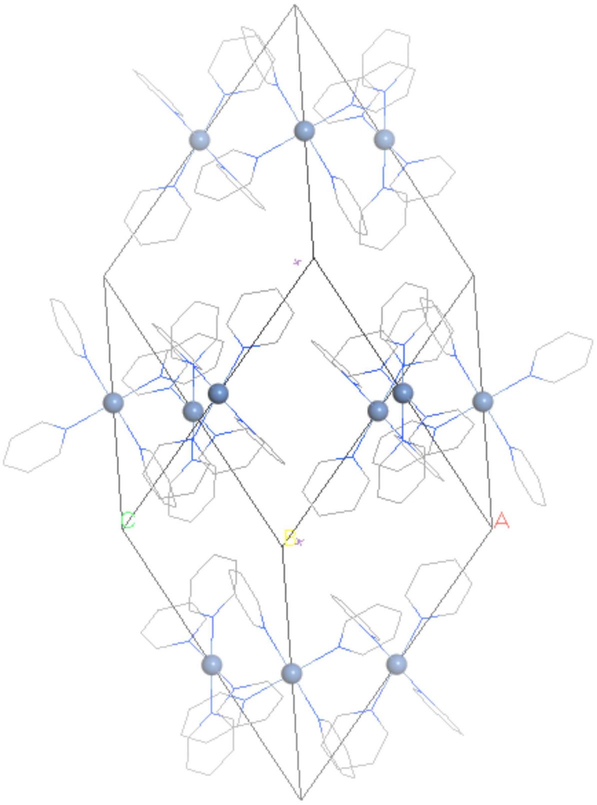

Figure S6: The computational unit cell is shown with the skeleton of the molecules illustrated. The Ni atoms are shown by blue spheres. The three Ni ions closest to the origin and the three Ni ions furthest from the origin form the lower and upper triangles considered in our schematic diagrams.

(a)

Structure	ΔE (meV)	ΔE (K)
out-of-plane, AFM	236	2740
out-of-plane, FM	202	2350
in-plane, AFM	17	200
in-plane, FM	0	0

(b)

Structure	ΔE (meV)	ΔE (K)
symmetric, in-plane AFMw	234	2730
asymmetric, in-plane AFMs	119	1360
symmetric, out-of-plane AFMw	105	1220
asymmetric, out-of-plane AFMs	0	0

Table 1: ΔE, the relative energy of a state, for (a) 3-link states; (b) the lowest lying magnetic configuration of each 2-link model.

Magnetic State	ΔE (meV)	ΔE (K)	Spin Density per cell ($\hbar/2$)	|Spin Density| per cell ($\hbar/2$)	|Mullikan spin population| per Ni ($\hbar/2$)
FM	0.54	6.3	6.00	6.79	1.53
AFMw	0.10	1.1	2.00	6.78	1.53
AFMs	0	0	2.00	6.78	1.53

Table 2: Relative energies ΔE of magnetic states for the lowest energy model (asymmetric, out-of-plane), integrated spin densities and average of the absolute value of the Mulliken spin population per Ni over the rhombohedral primitive cell.

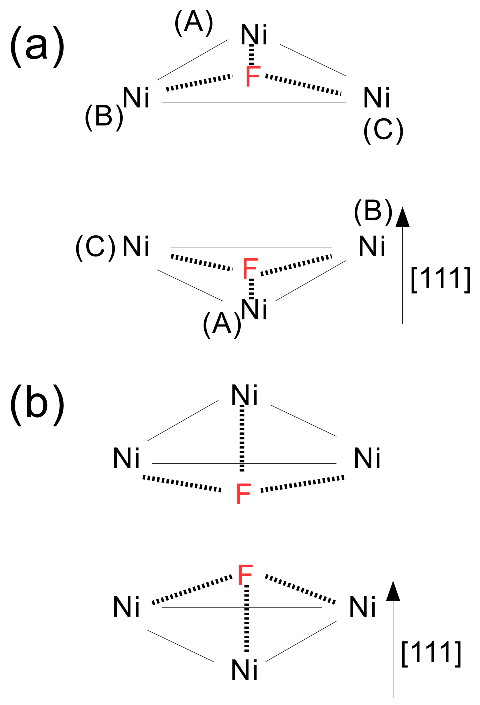

Figure S7: Position of the central F ion for the 3-link models relative to the upper and lower triangles of Ni in the rhombohedral cell (positions of other ions suppressed). (a) In-plane configuration, (b) out-of-plane configuration.

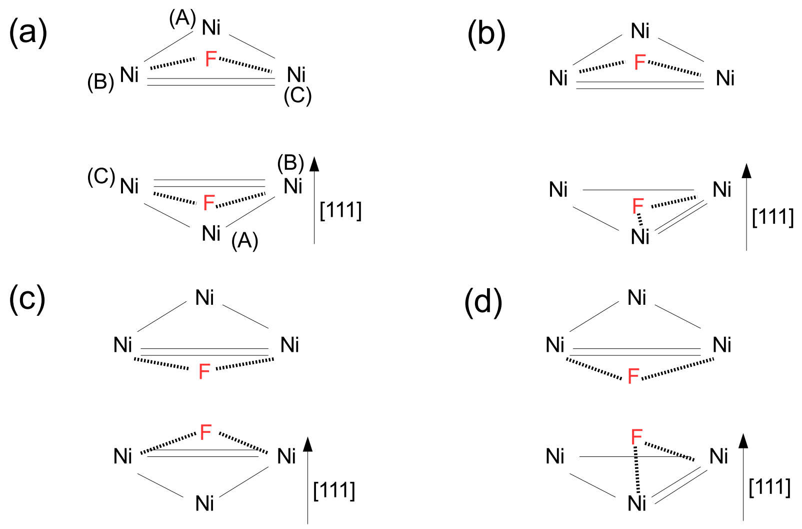

Figure S8: Schematic diagram of the links between Ni ions in the 2-link models. The structural configurations with the central F ion in roughly the same planes as the Ni triangles are (a) in-plane, symmetrical and (b) in-plane, asymmetrical; those with the F ions displaced towards the center of the cell are (c) out-of-plane, symmetrical; (d) out-of-plane, asymmetrical.

and the exchange constant

$$J = \frac{E_{\mathrm{AFM}} - E_{\mathrm{FM}}}{8}. \tag{4}$$

This is the value for the Ising exchange constant; in order to obtain the exchange constants for the Heisenberg model that are quoted in the main text we follow [6] and divide the Ising value by the number of magnetic sites in the cell, which is 3.

4.3.2 2-link asymmetric model

Starting with the Hamiltonian of the asymmetric Ising system

$$\hat{H} = -\sum_{\text{unit cells}} \left[J_{||} \left(\hat{S}_{\mathrm{A}}\hat{S}_{\mathrm{B}} + \hat{S}_{\mathrm{C}}\hat{S}_{\mathrm{B}} \right) + J_{|} \left(\hat{S}_{\mathrm{A}}\hat{S}_{\mathrm{B}} + 2\hat{S}_{\mathrm{A}}\hat{S}_{\mathrm{C}} + \hat{S}_{\mathrm{B}}\hat{S}_{\mathrm{C}} \right) \right], \tag{5}$$

we find the following values of the energy per unit cell for each state of interest:

$$E_{\mathrm{FM}} = -2J_{||} - 4J_{|}, \tag{6}$$
$$E_{\mathrm{AFMw}} = 2J_{|}, \tag{7}$$
$$E_{\mathrm{AFMs}} = 2J_{||}. \tag{8}$$

From these values we obtain the following simultaneous equations:

$$E_{\mathrm{FM}} - E_{\mathrm{AFMw}} = -2J_{||} - 6J_{|}, \tag{9}$$
$$E_{\mathrm{AFMs}} - E_{\mathrm{AFMw}} = 2J_{||} - 2J_{|}, \tag{10}$$
$$E_{\mathrm{FM}} - E_{\mathrm{AFMs}} = -4J_{||} - 4J_{|}. \tag{11}$$

This system is overdetermined, but as Eq. (11) is a linear combination of Eq. (9) and Eq. (10) it is possible to solve exactly to obtain the following for the exchange constants:

$$J_{||} = \frac{3E_{\mathrm{AFMs}} - 2E_{\mathrm{AFMw}} - E_{\mathrm{FM}}}{8}, \tag{12}$$
$$J_{|} = \frac{2E_{\mathrm{AFMw}} - E_{\mathrm{AFMs}} - E_{\mathrm{FM}}}{8}. \tag{13}$$

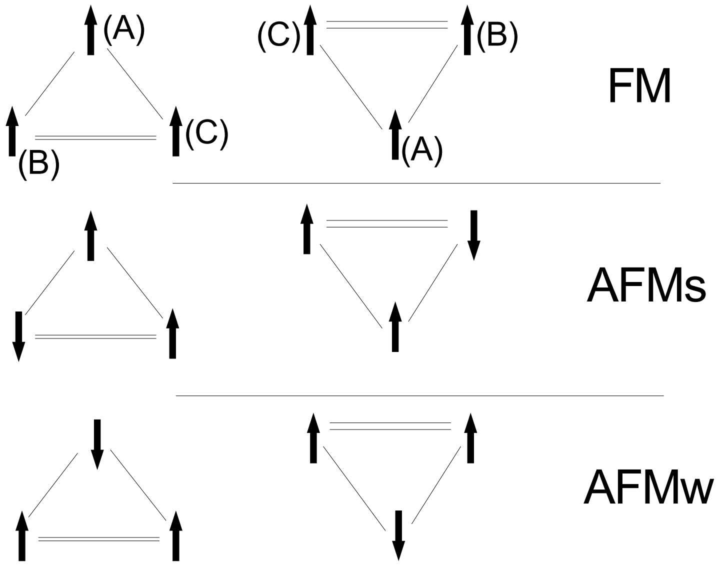

(a)

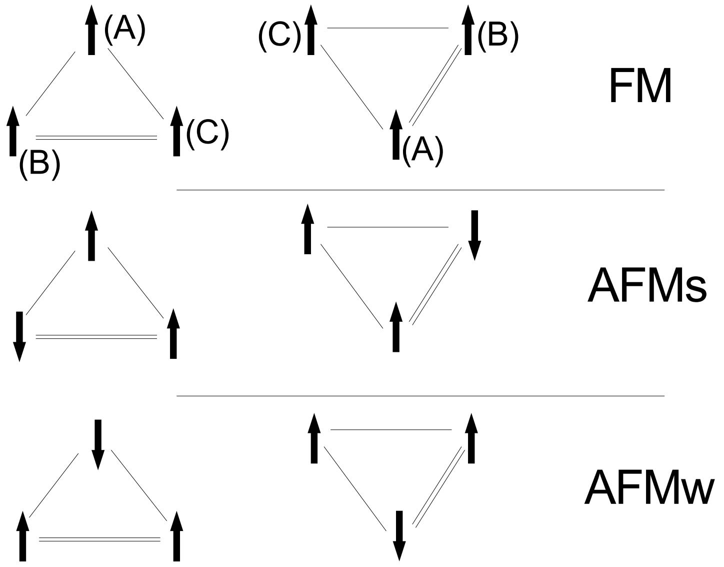

(b)

Figure S9: Possible magnetic states for (a) symmetric 2-link models, (b) asymmetric 2-link models.

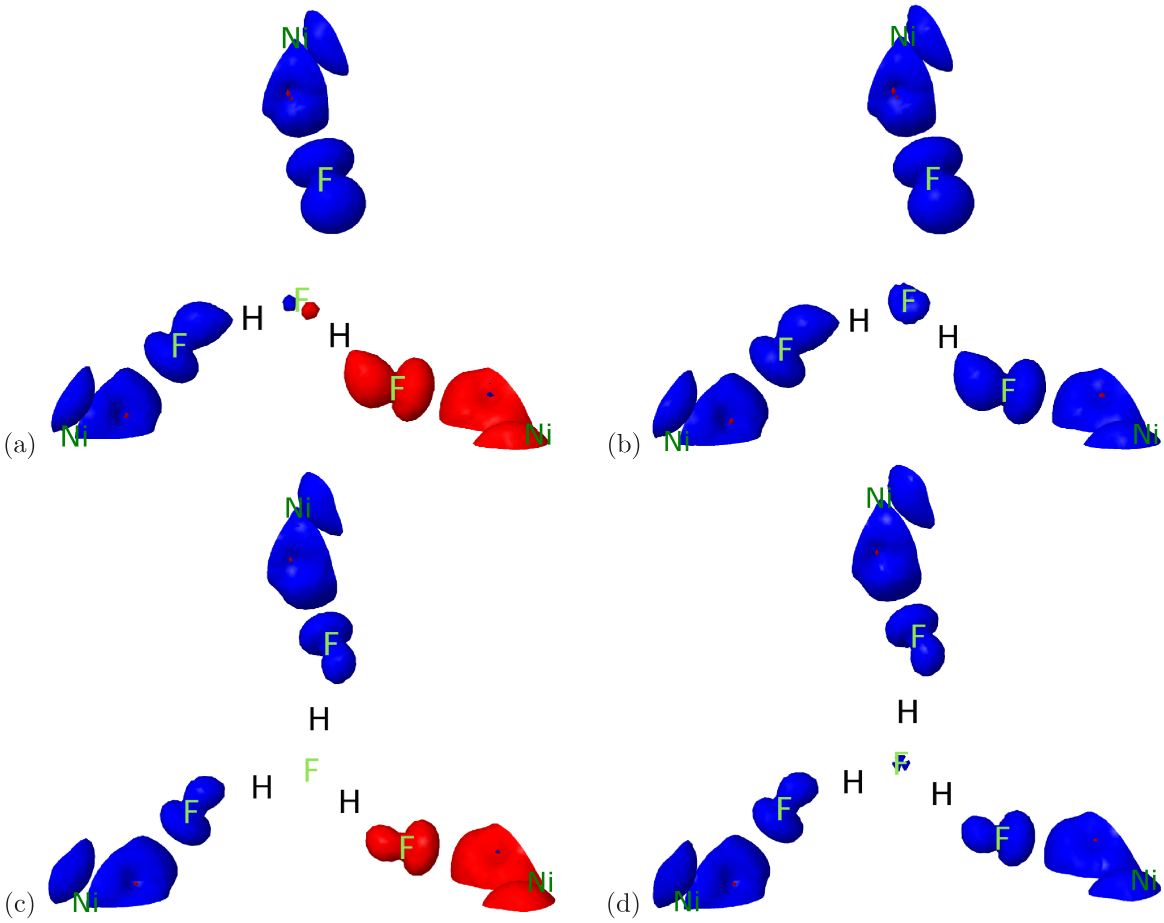

Figure S10: Spin isosurfaces at 0.01 a.u. for the moieties linking Ni ions in different magnetic configurations. (a) asymmetric, out-of-plane 2-link AFMs; (b) asymmetric, out-of-plane 2-link FM; (c) in-plane 3-link, AFM; (d) in-plane 3-link, FM.

Again, these are the values for the Ising exchange constants and so to obtain the Heisenberg values we follow [6] and divide by the number of magnetic sites in the cell, which is 3.

4.4 Spin Density Plots from Calculations

Fig. S10 shows plots of the spin density across the linking moieties of the Ni triangles for the in-plane 3-link and asymmetric, out-of-plane 2-link models. For the asymmetric, out-of-plane 2-link model we see for that both the AFMs and FM configurations [Fig. S10(a) and (b) respectively], the presence of the H ions has led to spin delocalisation from the F ions neighboring the Ni ions to the central F ion. In the AFMs case, we see two separate isosurfaces of opposite spin, and in the FM case a single isosurface of the majority spin. The F neighboring the unlinked Ni ion has a larger spin density. For the in-plane 3-link model, we see no spin density at the central F ion in the AFM case [Fig. S10(c)], which implies that any spin delocalisation there is negligible. In the FM case [Fig. S10(d)], we see a small, triple-lobed isosurface indicating that spin has been transferred from all three Ni-neighboring F ions to the central ion via the H ion links. Note also that the corresponding lobes are smaller for the in-plane 3-link configurations than the asymmetric, out-of-plane 2-link configurations. This is consistent with the unbroken network of superexchange that exists in the in-plane 2-link model: spin may delocalise between all Ni ions without interruption, whereas it only delocalises along links between members of the same trimer in the asymmetric, out-of-plane 2-link case.

References

[1] S. J. Clark, M. D. Segall, C. J. Pickars, P. J. Hasnip, M. J. Probert, K. Refson, and M. C. Payne, Z. Kristall. **220**, 567 (2005).

[2] K. Lejaeghere et al., Science **351**, doi:10.1126/science.aad3000.

[3] D. Vanderbilt, Phys Rev. B **41**, 7892 (1990).

[4] M.-H. Whangbo, H.-J. Koo, and D. Dai, Journal Solid State Chem. **176**, 417 (2003).

[5] I. de P. R. Moreira and F. Illas, Phys. Chem. Chem. Phys. **8**, 1645 (2006).

[6] S. N. Datta and N. Hansda, Chem. Phys. Lett. 621, 102 (2015).
